# Partial Resection of a Reconstruction Plate After Mandibular Reconstruction Using a Free Fibula Osteocutaneous Flap: Another Approach to Keep It Simple

**Published:** 2018-10-05

**Authors:** Luke T. Meredith, Bradley J. Vivace, Thomas J. Lee, Bradon J. Wilhelmi

**Affiliations:** ^a^University of Louisville School of Medicine, Louisville, Ky; ^b^Department of Plastic and Reconstructive Surgery, University of Louisville, Ky

**Keywords:** mandibular reconstruction, partial plate resection, orthognathic surgery, reconstructive plate complications, osteocutaneous flap

## DESCRIPTION

This case involved partial resection of a Stryker reconstruction plate 10 years after mandibular reconstruction due to squamous cell carcinoma. The plate was removed because of fistula formation due to plate extrusion. Large wire cutters were used to partially remove the plate to avoid unnecessary injury to the tissue.

## QUESTIONS

1. What is the relevant medical and surgical history of this patient?
2. Why is this procedure more complex in a patient who has undergone radiation therapy?
3. What was the procedure involved in this case?
4. Why was it advantageous to use large wire cutters to partially remove the plate?

## DISCUSSION

**Figure 1:**
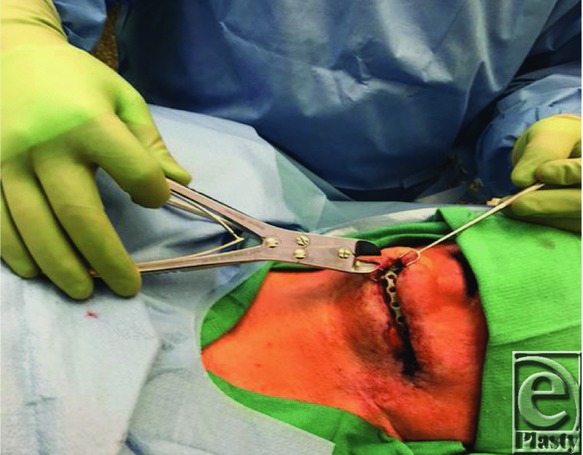
Intraoperative: large wire cutter and problematic segment of the reconstruction plate.

**Figure 2:**
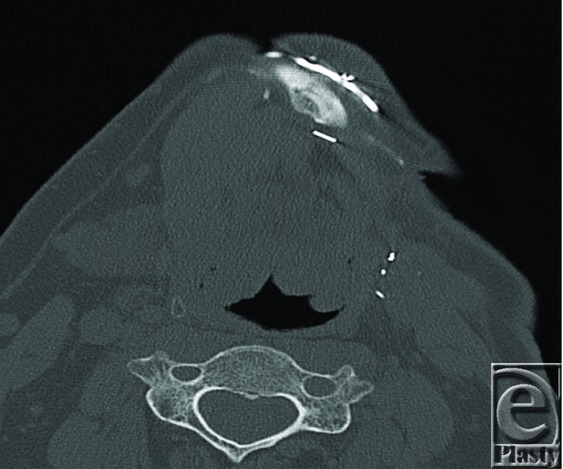
Computed tomographic scan showing problematic region of the plate.

This case involves the partial removal of a mandibular reconstruction plate in a 55-year-old male patient. Reconstruction of this patient's left jaw structure was carried out after a composite mandibulectomy due to squamous cell carcinoma. A free fibula osteocutaneous flap was used to reconstruct the mandible and the floor of the mouth. The osteosynthesis involved the use of a Stryker reconstruction plate to secure the fibula bone graft into the defect. The patient did well for 10 years with no problems. Subsequently, 3 fistulas formed because of plate extrusion, leading to chronic infection and persistent draining. Figures 1 and 2 show the problematic segment of the plate prior to removal.

Hardware-associated complication rates are approximately 15% in mandibular reconstruction, with the most common being plate extrusion.^1^ This risk is associated with patients who have previously undergone radiation therapy. Radiation can cause significant damage to the involved area of tissue, with long-term effects including decreased vascularity, in addition to hypoxia.^2^ In the involved area of skin, the predominating long-term damage is a result of cutaneous and subcutaneous fibrosis.^2^ Importantly, the changes in morphology that can be seen in tissue damaged by radiation include vessels that are dysmorphic and occluded, with damaged endothelium leading to decreased perfusion.^3^ Therefore, any surgical procedure that involves previously irradiated tissue and skin will need to take steps to minimize tissue damage to attain a desirable outcome.

**Figure 3:**
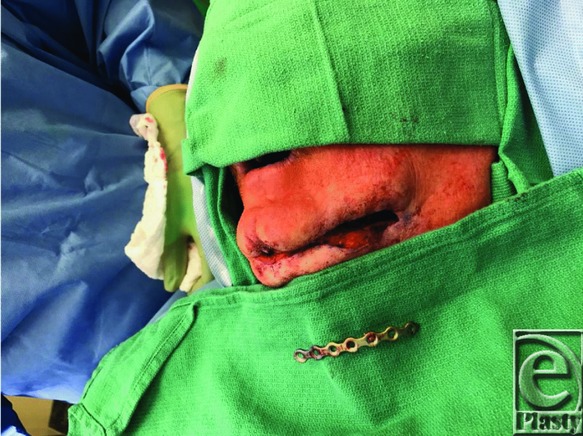
Intraoperative: Plate removed.

**Figure 4:**
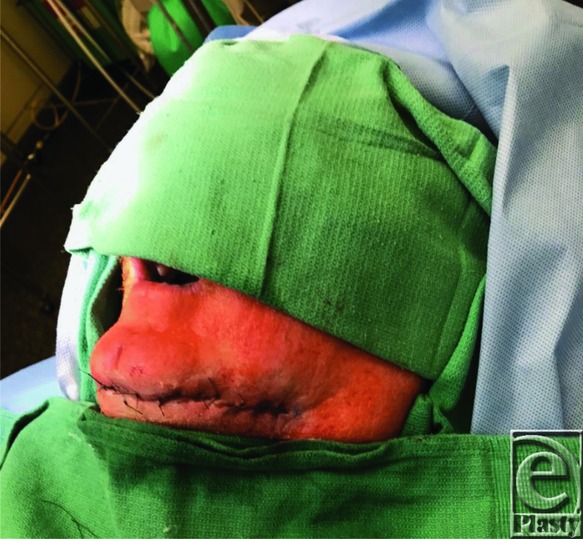
Wound closure.

The procedure used in this case is now discussed. To minimize damage to the tissue, the operation focused solely on the region of the plate where the 3 fistulas were present. The defects were communicated per one wound. Then, the problematic region of plate and screws was dissected superiorly and inferiorly, entirely exposing the problematic segment. Next, the screws fastening this segment of plate to the bone were removed. Segmental resection of the plate was then accomplished using a Stryker large wire cutter to cut through the plate on both sides of the defective area, freeing the segment. Bolt cutters were initially used in the attempt to remove the plate, as in a previous case at our institution reported by Boyd et al.^4^ However, the cutting edge of the bolt cutter was unable to efface the plate in this case. The use of a large wire cutter was then attempted and successfully allowed for a very controlled removal of the problematic segment. Figures 3 and 4 show the removal of the plate and wound closure.

The reconstruction plates used for bone flaps can be long, and the exposure needed to remove the entire plate is extensive and undesirable when the adjacent soft tissue has been damaged by radiation. The ability to perform a partial excision of the plate in this operation allowed for a smaller incision, causing minimal tissue damage. Segmental resection was also advantageous because removal of the plate up to the angle of the mandible increases the risk of facial nerve injury. Because the most anterior part of the plate was unproblematic, leaving it in place allowed further scarring on the face to be avoided. The patient is now 6 months post-operation, with no problems and a well-healed wound.

The aforementioned case involved the partial resection of a reconstruction plate using a Stryker large wire cutter. This method of removal was used because of the patient's history of radiation therapy in that region for the treatment of squamous cell carcinoma. Partial removal decreased the risk of complications and unnecessary facial scarring. The use of large surgical wire cutters allowed for the controlled partial removal of the plate with minimal injury to the fragile tissue.
